# Aquareovirus protein VP6 colocalizes with NS80 protein in infected and transfected cells

**DOI:** 10.1186/1743-422X-10-133

**Published:** 2013-04-27

**Authors:** Dawei Wen, Liming Yan, Ling Shao, Hong Guo, Xiaoming Li, Qin Fang

**Affiliations:** 1 State Key Laboratory of Virology, Wuhan Institute of Virology, Chinese Academy of Sciences, Wuhan, 430071, China; 2 University of the Chinese Academy of Sciences, Beijing, 100039, China; 3 School of Basic Medical Sciences, Wuhan University, Wuhan, 430072, China

**Keywords:** Aquareovirus, Structural protein VP6, NS80, Colocalization

## Abstract

**Background:**

Aquareovirus particle is comprised of central core and outer capsid, which is built by seven structural proteins (VP1-VP7). The protein VP6 has been identified to be a clamp protein of stabilizing inner core frame VP3, and bridging outer shell protein VP5. However, the biological properties of VP6 in viral life cycle remain unknown.

**Results:**

The recombinant VP6 (rVP6) of aquareovirus was expressed in *E. coli*, and the polyclonal antibody against VP6 was generated by using purified rVP6 in this study. Following the preparation of VP6 antibody, the VP6 component in aquareovirus infected cells and purified viral particles was detected by Immunoblotting (IB) assay. Furthermore, using Immunofluorescence (IF) microscopy, singly transfected VP6 protein was observed to exhibit a diffuse distribution mainly in the cytoplasm, while it appeared inclusion phenotype in infected cells. Meanwhile, inclusion structures were also identified when VP6 was coexpressed with nonstructural protein NS80 in cotransfected cells.

**Conclusions:**

VP6 can be recruited by NS80 to its inclusions in both infected and transfected cells. The colocalization of VP6 and NS80 is corresponding to their homologous proteins σ2 and μNS in MRV. Our results suggest that VP6 may play a significant role in viral replication and particle assembly.

## Background

Aquareovirus, one of the members in the *Reoviridae* family, can infect aquatic animals including bony fish, and shellfish [1]. Many of these viruses have been isolated from fresh or seawater in the past years [2,3]. It has been recognized that grass carp reovirus (GCRV), which is classified to species *Aquareovirus*-C in the genus *Aquareovirus*, is the most pathogenic aquareovirus among all the isolates reported to date [2].

Similar to other members in the family of *Reoviridae*, aquareoviruses are nonenveloped, multishelled particles that display icosahedral symmetry with an overall diameter of approximately 75–80 nm [4,5]. Phylogenic analysis and three-dimensional (3D) image reconstructions of aquareoviruses by cryo-electron microscopy (Cryo-EM) indicated that the similarities between aquareoviruses and orthoreoviruses in their protein sequences are accordant to their particle organization [4-8]. Studies on genome sequences revealed that the 11 segmented dsRNA genome of aquareovirus encoded twelve unique viral proteins [4-7]. Seven structural proteins (VP1-VP7) compose the viral particle including outer capsid and central core [5]. The outer capsid of the virion is made up of VP5 and VP7, which might be related to virus entry and interaction between virus and host cells during infection. The inner shell frame is built by proteins VP3 and VP6, and the viral core also contains the RNA polymerase complex (proteins VP1-VP3 and VP4) [5,6]. The core may play a role responsible for viral replication. In addition, five nonstructural proteins that are not assembled into viral particles also perform functions in replication cycles same as other reoviruses.

The aquareovirus protein VP6, encoded by segment S8, consists of 412 amino acids (45 kDa) [7]. Recent studies on single particle reconstruction revealed that VP6 protein occupies very special location in virus particle construction, which presented 120 copies in each virus particle [6,8,9]. This is very similar with the organization observed in Cypovirus (CPV), but different from that of the mammalian orthoreoviruses (MRV) and avian orthoreoviruses (ARV) cores where 150 protruding molecules were found [1,10]. In addition, bioinformatics analysis indicated that aquareovirus VP6 and MRV σ2 share the amino acid sequence identity ranging from 20% to 22%. Although there is high sequence conservation in their C-terminals, and the conserved sequence distributions are across most of the protein lengths including their similar secondary structure prediction profiles [11]. In MRV, σ2 is reported to be required for forming stable core-like particles with λ1, and/or as a clamp for stabilizing the λl shell [11,12]. In this regards, it is suggested that VP6 may play similar role as σ2 in MRV during viral replication and assembly.

Reovirus replication and assembly are thought to occur within viroplasms or viral inclusions that form in the cytoplasm of infected cells [13,14]. Similar to other reoviruses, aquareovirus replication and assembly occur in cytoplasmic inclusion structures, and the NS80 protein has been demonstrated to play critical role in inclusion formation in both infected and transfected cells [15,16]. Previous investigation in our lab identified that there is an interaction between NS80 and VP6 by the yeast two-hybrid (Y2H) system [17]. In addition, the nonstructural protein NS80 was verified to be essential for viral cytoplasmic inclusion structure formation, and it can recruit NS38 and minor core protein VP4 to its inclusions [15,16]. However, the biological properties of VP6 involved in inclusion formation or viral replication remain unknown.

To characterize aquareovirus VP6 and define its relationship with NS80 protein in virus replication and assembly, we currently investigated the biological properties of VP6 in both infected and transfected cells in this study. We detected that VP6 protein exhibited a diffuse distribution in singly transfected cells utilizing Immunofluorescence (IF) assay, while it appeared inclusion phenotype in infected cells. Again, we also identified inclusion like structures when VP6 was cotransfected with nonstructural protein NS80. The evidence that NS80 recruited VP6 in cotransfected cells and the both proteins co-localization suggest that VP6 may be recruited by NS80 in infected cells. The data provide a reliable evidence for further study of the VP6 function during aquareovirus replication and assembly.

## Results

### Expression and purification of VP6 *in vitro*

**Figure 1:**
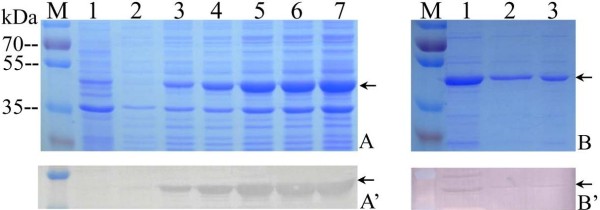
**Identification and purification of recombinant VP6 protein *in vitro*. A**: SDS-PAGE analysis of induced recombinant VP6 expression. M, standard protein marker; Lane 1, expression of pREST empty vector induced by IPTG for 3 h; 2–7, expressed recombinant VP6 cell lysate pellet induced by IPTG for 0, 1, 2, 3, 4, 5 h respectively. **A’**: Western blotting analysis corresponding to lane 1–7 in A with His-tag monoclonal antibody. **B**: SDS-PAGE analysis of the purified rVP6 protein. Lane 1, recombinant VP6 cell lysate; Lane 2, 3, purified rVP6. **B’**: Western blot analysis of purified rVP6 protein matching lane 1–3 in B with rabbit anti-GCRV antibody. Arrows indicate rVP6 protein.

SDS-PAGE analysis of recombinant VP6 (rVP6) protein expression *in vitro* showed that the time course expression of rVP6 appeared gradually increased by IPTG inducing at 1, 2, 3, 4, 5 h respectively. The His-tag fusion VP6 protein was expressed correctly with a molecular mass of about 48 kDa as shown in Figure 1A, which was consistent with rVP6 predicted size because the increased 3 kDa is related to N-terminal tag in pRSET vector. Further IB analysis indicated that the expressed fusion protein rVP6 was able to bind immunologically to anti-His-tag monoclonal antibody (Figure 1A’), suggesting that rVP6 protein was induced by IPTG, and the expressed product is the interest fusion protein. Given that the expressed protein is His-tagged fusion protein, Ni^2+^-Chelating resin column was used in the further purification of the fusion protein. As shown in Figure 1B, the purified rVP6 appeared nearly single band corresponding to the molecular weight of the interest protein in comparison with unpurified cell lysates. Furthermore, the purified rVP6 protein and its cell lysate could react immunologically with GCRV polyclonal antibody (Figure 1B and 1B’), implying that the recombinant VP6 fusion protein is GCRV related antigen that belongs to viral structural proteins. The above SDS-PAGE and IB analyses showed that VP6 protein was induced by IPTG, and the results also indicated the purified rVP6 is qualified for antibody preparation.

### Detection of the VP6 protein in GCRV infected CIK cell lysate

**Figure 2:**
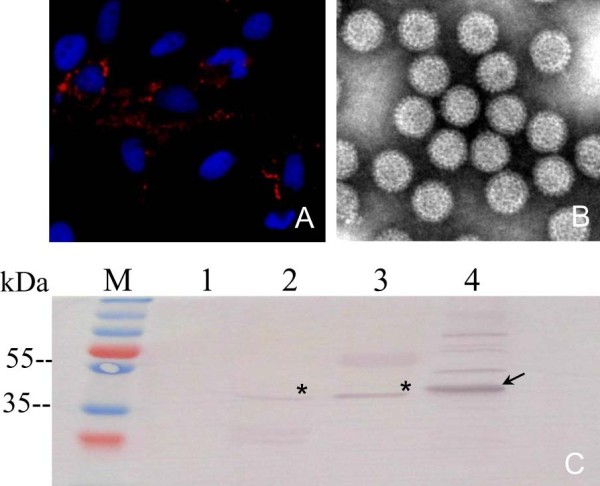
**Detection of the VP6 protein in virus infected cells. A**: The VP6 protein was detected in GCRV infected CIK cells by immunostaining with mouse anti-VP6 polyclonal serum followed by Alexa Fluor® 568 donkey anti-mouse IgG (red). **B**: Purified GCRV particles observed by TEM. **C**: Western blotting analysis of the VP6 protein in virion and infected CIK cells using mouse anti-VP6 polyclonal serum. M, standard protein marker; Lane1, mock-infected CIK cell lysates; Lane 2, GCRV infected CIK cell lysates; Lane 3, purified GCRV particles; Lane 4, recombinant His-tag fusion VP6 protein; Asterisk(*) indicates VP6 (45 kDa). Arrow presents rVP6 protein (48 kDa).

Subsequently, we performed experiments to detect VP6 protein in aquareovirus infected cells by using prepared mouse anti-VP6 polyclonal antibody. Indirect IF examination showed that VP6 protein in viral infected cells could be detected with VP6 antibody. As shown in Figure 2A, VP6 expression in infected cells presented to distribute mainly in the cytoplasm with punctuate appearance, whereas no fluorescence presented in mock infected cells (IF data not shown), indicating that the VP6 antibody can immunologically recognize VP6 protein in virus infected cells. To verify if the antibody is specific to VP6 protein in aquareovirus particles, we purified virion from infected cell culture supernatant. The transmission electron microscopy image, as shown in Figure 2B, indicated that viral particles were highly purified. Further IB analysis was also conducted with infected cell lysates and purified virus particles as well as rVP6 as positive control. Following result showed a specific immunological band crossing with aquareovirus infected cell lysates and purified virion with molecular weight at about 45 kDa. The target band of His-tag fusion rVP6 is a little higher than that of original VP6, which is corresponding to the expected size of rVP6 (Figure 2C). No visible band could be detected with the mock infected CIK cells.

### Single VP6 expression is diffusely distributed in transfected cells in the absence of other viral proteins

**Figure 3:**
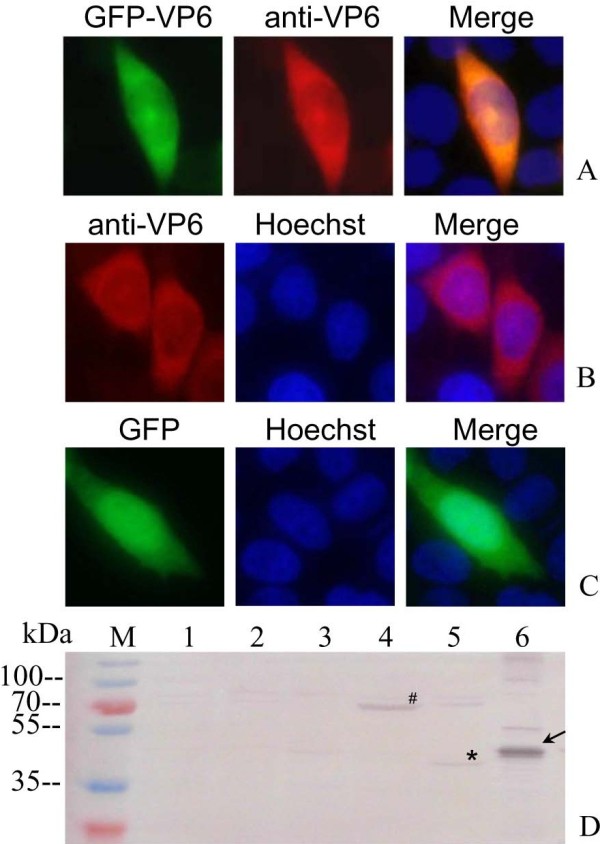
**Indirect IF and IB assays of the VP6 protein in transfected cells. A**, **B** and **C**: Intracellular localization of single VP6 protein in transfected cells, Vero cells were transfected with plasmid pEGFP-VP6 (**A**), pCI-VP6 (**B**) and pEGFP (**C**) respectively for 24 h, and then immunostained with mouse anti-VP6 polyclonal serum followed by Alexa Fluor® 568 donkey anti-mouse IgG (red) for EGFP-VP6 and VP6. The nuclei were visualized by counterstaining with Hoechst. **D**: IB analysis of expression of pEGFP-VP6 and pCI-VP6 in transfected Vero cells. M, standard protein marker; Lane1, mock transfected Vero cell lysates; 2–5, transfected Vero cell lysates with pCI-neo empty vector, pEGFP-C1 empty vector, pEGFP-VP6 (71 kDa) plasmid, pCI-VP6 (45 kDa) plasmid respectively; Lane 6, recombinant His-tag fusion VP6 protein (48 kDa) as positive control. #: GFP-VP6, *: VP6, Arrow presents rVP6 protein.

To understand the nature of single VP6 expression, we investigated the intracellular distribution of single VP6 expression in transfected cells. For this purpose, the GFP-tagged VP6 (pEGFP-VP6), and non-tagged VP6 (pCI-VP6) recombinants were transfected into Vero cells respectively, and pEGFP vector was used as control in this experiment. Immunofluorescence results indicated that the GFP-fused VP6 protein expressed in transfected cells was mainly diffusely distributed in the cytoplasm, and the dispersed phenotype was confirmed by non-fusion VP6 expression in pCI-VP6 transfected cells (Figure 3A and 3B). As control, the GFP was diffusely distributed throughout all the cells including cytoplasm and nucleus (Figure 3C). Apparently, the phenotype of singly expressed VP6 in transfected cells appeared different from its distribution in viral infected cells. To further determine the correctness of recombinant protein expressed in transfected cells, all the cell lysates were subjected to SDS-PAGE, followed by immunoblotting with mouse anti-VP6 polyclonal serum. The IB result, as shown in Figure 3D, identified that the expression of GFP-tagged VP6 and non-tagged VP6 proteins in transfected cells produced specific immunological bands with VP6 antibody. The corresponding molecular weight is about 71 kDa for GFP-VP6 and 45 kDa for VP6 respectively, which were consistent with their predicted protein value, and no specific band was detected with the GFP and mock transfected cells. The results verified that all the recombinants were correctly expressed in transfected cells.

### VP6 colocalized with NS80 in virus infected cells

**Figure 4:**
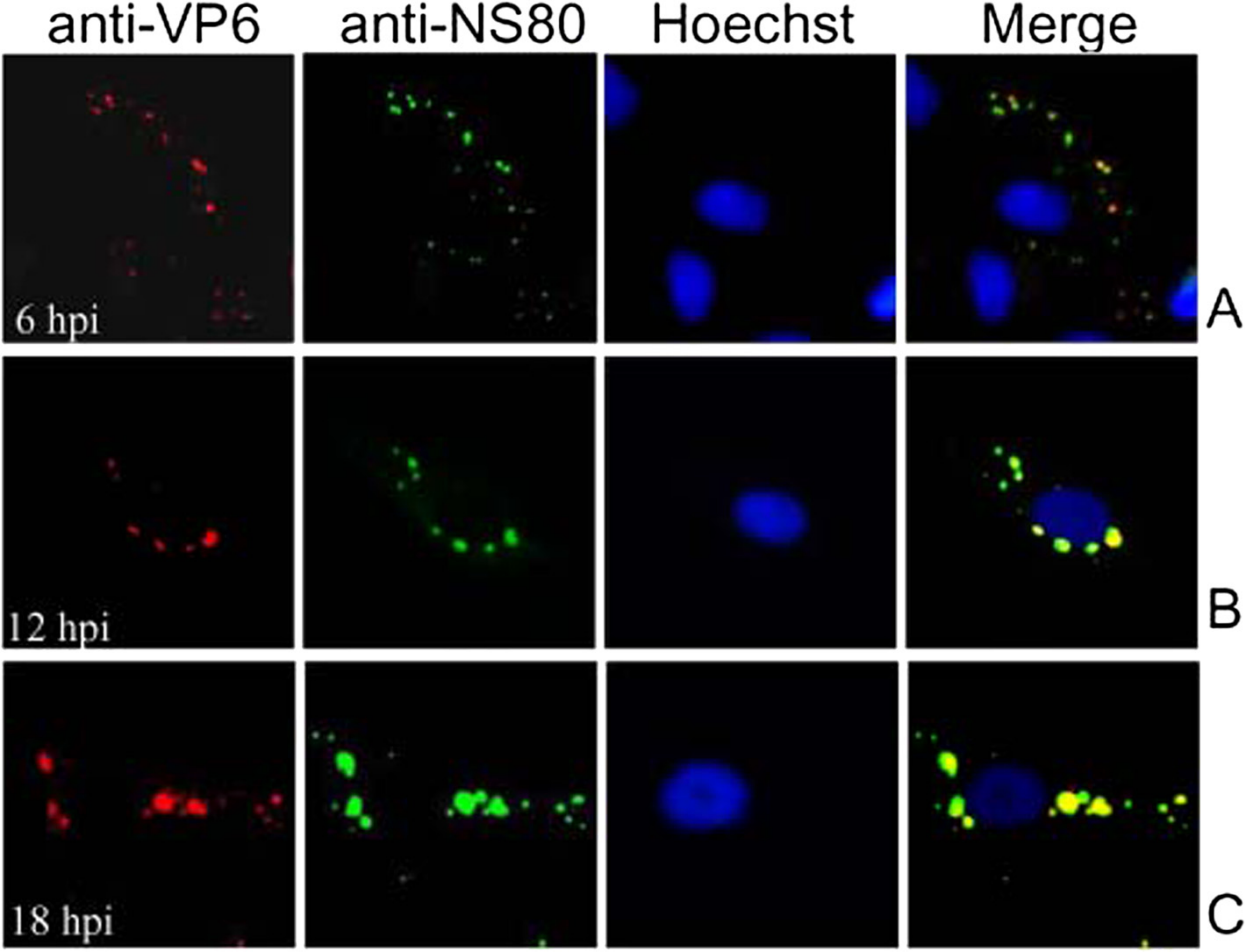
**Subcellular colocalization of the VP6 and NS80 proteins in virus infected cells.** IF microscopy of GCRV infected CIK cells at 6 h (**A**), 12 h (**B**), 18 h (**C**), p.i. The subcellular localizations of VP6 and NS80 were detected by immunostaining with mouse anti-VP6 and rabbit anti-NS80 polyclonal antibodies followed by Alexa Fluor® 568 donkey anti-mouse IgG (red) and Alexa Fluor® 488 donkey anti-rabbit IgG (Green). Nuclei were counterstained with Hoechst (blue).

To identify if there is a colocalization between VP6 and nonstructural protein NS80 in virus infected cells, we examined the subcellular localization of NS80 as well as VP6 by immunostaining with specific antibodies (anti-VP6 and anti-NS80 polycloncal antibodies) at different times post infection. Following results revealed that VP6 and NS80 were first detectable at 6 h p.i., which appeared at the same location as small immunofluorescence spots that scattered in the cytoplasm (Figure 4A). As infection progressed, VP6 and NS80 remained in the inclusions with obvious colocalization at 12 and 18 h p.i. (Figure 4B and 4C), while no VP6 and NS80 expression was detected in mock infected cells (data not shown). These results confirmed that VP6 has colocalization with NS80 in virus infected cells.

### VP6 localized to globular inclusions when coexpressed with NS80 in transfected cells

**Figure 5:**
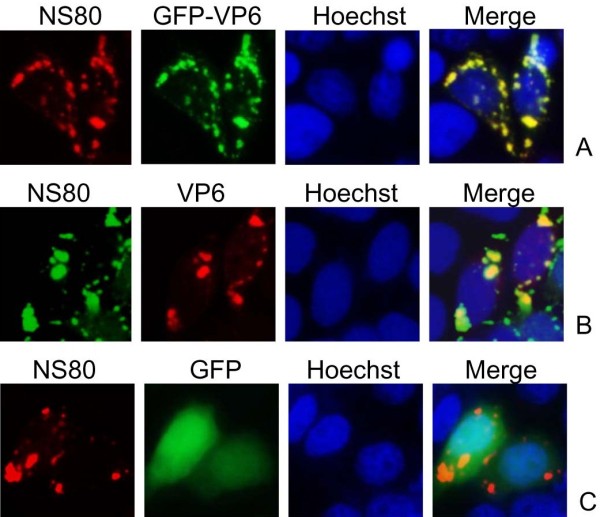
**Subcellular colocalization of the VP6 and NS80 proteins in cotransfected cells.** IF microscopy of Vero cells cotransfected with plasmids pCI-NS80 and pEGFP-VP6 (**A**), pCI-VP6 (**B**), pEGFP-C1 (**C**) for 20 h. The intracellular localizations of NS80 and VP6 were detected by immunostaining with rabbit anti-NS80 followed by Alexa Fluor® 488 donkey anti-rabbit IgG (Green, **B**) or Alexa Fluor® 568 donkey anti-mouse IgG(red, **A** and **C**), and mouse anti-VP6 polyclonal antibodies followed by Alexa Fluor® 568 donkey anti-mouse IgG (red, **B**). The nuclei were visualized by counterstaining with Hoechst (blue).

To determine whether NS80 could alter the dispersed phenotype of VP6 in singly transfected cells, we performed experiments to cotransfect the plasmids expressing GFP, GFP-VP6, and VP6 with pCI-NS80 recombinant respectively into Vero cells, and conducted IF analysis. As shown in Figure 5A, the inclusion structures appeared when GFP-tagged VP6 cotransfected with NS80, which was clearly different from that of GFP-VP6 protein expressed solely. Besides, when VP6 and NS80 were expressed in cotransfected cells, as expected, the VP6 and NS80 proteins were observed to colocalize in cytoplasmic inclusions (Figure 5B), while GFP could not colocalize with NS80 in transfected cells (Figure 5C). Taking together, the results indicated both GFP-VP6 and VP6 could colocalize with NS80, demonstrating that VP6 can be recruited by NS80 to its inclusions.

## Discussion

Reovirus factories were determined to contain fully and partially assembled viral particles, viral proteins, double-stranded RNA, and cell factors [18,19]. It has been recognized that these multiplex modules are highly organized dynamic spacial structures that support viral genome replication and particle assembly [20,21]. As a structural protein of aquareovirus, VP6 exhibited as nodules on the surface of inner capsid, and also known as the counterpart of protein σ2 in MRV [11]. It suggests that VP6 may play a role in viral inclusion formation and particle assembly.

Previously, we demonstrated that the NS80 was able to form inclusions in both infected and transfected cells, and also could recruit NS38 or VP4 to its inclusion. In this study, we detected that VP6 expression appeared as inclusion phenotype in the cytoplasm in virus infected cells by IF, and the correct expression of VP6 was also confirmed utilizing IB assay. In addition, time course infection experiments indicated that colocalization of VP6 and NS80 could be initially detected at 6 h post infection. As infection progressed, VP6 and NS80 remained in the inclusions as they grew in size and moved to the perinuclear region in infected cells. Further cotransfection with VP6 and NS80 expressing plasmids indicated that VP6 colocalized with NS80 in inclusion structures, which was totally in contrast to diffused distribution pattern when VP6 was singly expressed in transfected cells, suggesting that VP6 could be recruited by NS80 to inclusions. These inclusion structures resembled the globular phase-dense viral factories in infected CIK cells. These results proved that NS80 can recruit VP6 to its inclusions and supported our previous conclusion that NS80 can recruit viral proteins to its inclusions [16].

It may need to note that VP6 was found to colocalize with NS80 in cytoplasm in both transfected and virus infected cells in this study. This result is corresponding to the colocalization of their homologous proteins σ2 and μNS in MRV [22], but different from σA and μNS in ARV because σA can not be recruited by μNS to its inclusions [23]. As previously reported, no enzymatic activity has been ascribed to the core nodule protein σ2 in MRV. The significance of a dsRNA-binding activity of σ2 remains unknown [24], but a comparable activity of the ARV homolog σA has been shown to play a role in combating cellular antiviral responses [25-27]. This suggests that there may be a different mechanism between aquareovirus and ARV. In addition, intracellular post-translational cleavage of σA was found in ARV [28], but not observed in σ2 of MRV or in VP6 of aquareovirus, hinting that VP6 is more close to MRV σ2 in some biological properties. The results of VP6 colocalization with NS80 in cells suggest that the core protein component VP6 might be expressed and assembled in viral factory of the cytoplasm, similar to the σ2 and μNS in MRV [22]. However, the dsRNA binding or other properties of VP6 involved in viral replication events need to be further defined.

The recombinant VP6 was expressed in prokaryotic cells and the polyclonal anti-VP6 antibody was generated utilizing purified rVP6. Following the preparation of VP6 polyclonal antibody, the VP6 protein was detected in infected cell lysates and purified particles. In addition, we identified that the singly expressed VP6 appeared diffused distribution mainly in the cytoplasm in transfected cells. However, the expression of VP6 in aquareovirus infected CIK cells was shown in inclusion-like structures, which was confirmed by coexpressing VP6 with NS80 protein in cotransfected Vero cells. Our results provide a reliable evidence for further studying the VP6 function during aquareovirus replication and assembly.

## Conclusion

The results provided in this study indicate that VP6, which is a component of the aquareovirus particle, colocalized with NS80 in cotransfected as well as in infected cells, suggesting that VP6 can be recruited by NS80 to its inclusions. Our results laid a foundation for further studies aimed at understanding the function of VP6 in viral replication and particle assembly.

## Methods

### Cells and virus

CIK (*Ctenopharyngodon idellus* kidney) cells were used for viral infection, and Vero cells were prepared for cell transfection in this study. The CIK and Vero cells were cultivated in Eagle’s minimum essential medium (Eagle’s MEM, Invitrogen, USA), and Dulbecco’s Modification of Eagle’s Medium (DMEM, Invitrogen, USA) supplemented with 10% of fetal bovine serum (FBS), respectively. The original strain of aquareovirus-C GCRV-873, isolated and stored at author’s laboratory [29,30], was used in this study.

### Reagents and antibodies

T7 expression system (pRSET vector with BL21 (DE3) pLysS, and ProBond Resin) used for recombinant protein expression and purification plus Lipofectamine 2000 for transfection were the products of Invitrogen (Invitrogen, Carlsbad, USA). pCI-neo vector was purchased from Promega Co. (Promega USA). pEGFP-C1 vector was the product of Clontech Co. (Clontech,USA). All restriction enzymes were obtained from Takara Bio Inc. (Takara, Dalian, China) unless otherwise stated.

Rabbit or mouse polyclonal antibodies against GCRV-873 and NS80 were raised in our laboratory as reported previously [15,16,31]. His-tag monoclonal antibody was the product of Santa Cruz Biotechnology, inc. Alexa Fluor® 568 donkey anti-mouse IgG(H+L) (Red) and Alexa Fluor® 488 donkey anti-rabbit IgG(H+L) (Green) were purchased from Invitrogen Co. (Invitrogen, Carlsbad, USA).

### Recombinant plasmid constructions

To generate the recombinant that expresses VP6 in pRSET vector, the primers of S8 segment were designed based on GenBank sequences (AF403394), and restriction enzyme digestion sites were introduced at 5′ end of each primer pairs. The sense primer was: 5’CATGGATCCATGGCACAGCGTCAGTTT 3’(*Bam*H I underlined) and the antisense primer was: 5’GCTAAGCTTTTAGACGAACATCGCCTG3’(*Hind* III underlined). For the expression of VP6 in eukaryotic cells, the S8 gene was cloned into pCI-neo vector. The sense primer was: CATGAATTCATTTTGTGATGGCACAGCGTC3’ (*EcoR* I underlined) and the antisense primer: 5’ GCTTCTAGACAGTTAGACGAACATCGCCTG3’. (*Xba* I underlined). The pEGFP-C1 vector was also used to generate construct for the expression of *Aequorea victoria* enhanced green fluorescence protein (GFP) fusing to the N-terminus of the VP6 protein. The NS80 recombinant used in this study was previously described [15,16]. The correctness of the constructed recombinants was assessed by using regular enzyme digestion and plasmid sequencing (Invitrogen Biotechnology Inc, Shanghai, China).

### Expression of recombinant VP6 and antiserum preparation

To express rVP6 in *E. coli*, the positive recombinant transformant was grown in SOB medium as described previously [15]. After being induced by IPTG for 1 h, 2 h, 3 h, 4 h, 5 h at 28°C, all the lysate extracts of expressed bacteria were resuspended in phosphate-buffered saline (PBS), and stored at −30°C for further analysis. The purification of His-tag fused rVP6 protein was performed according to the ProBond™ Resin kit instruction. The preparation of VP6 polyclonal antibody either in New Zealand white rabbits or BALB/C mice was performed according to regular method in our lab as described previously [31]. The entire protocol and the animal experiments were approved by the Ethics Committee of Wuhan Institute of Virology, CAS.

### Infection, virus purification and transmission electron microscopy (TEM)

To carry out infection assay, CIK monolayers were infected with aquareoviruses at a multiplicity of infection (MOI) of 5 PFU/cell. Following 30 min of adsorption, cells were washed with 1xPBS to remove the inoculums, and fresh medium supplemented with 2% of fetal bovine serum (MEM-2) was added for viral propagation at 28°C. The virus-infected CIK cells could be fixed for Immunofluorescence (IF) assay when the cytopathic effects (CPE) were observed. In addition, the rest viral infected cell supernatants were collected at 48h post-infection (p.i.) for further viral particle purification. The intact virion was isolated by using CsCl density gradient centrifugation, and purified virus particles were examined under transmission electron microscope (Hitachi 7000-FA) as described elsewhere [32].

### SDS-PAGE and Immunoblotting analyses

For protein identification and immunoblotting (IB) analyses, cell lysates were collected, pelleted and resuspended in PBS, and then resolved by 10% sodium docecyl sulfate - polyacrylamide gel electrophoresis (SDS-PAGE). All samples in gels were transferred to polyvinylidene fluoride (PVDF) membranes by a semi-dry transfer cell (Bio-Rad, California, USA) for 45 min at 100 mA. As usual, resulting protein bands were detected by developing with nitrobluetetrazolium (NBT) /5-bromo,4-chloro,3-indolylphosphate (BCIP) alkaline phosphatase (AP) substrate solution according to the methods as described previously [16,31].

### Transfection and Immunofluorescence microscopy

The recombinant pEGFP-VP6, pCI-VP6 and pCI-NS80 plasmids as well as control plasmid pEGFP were used for either single transfection or cotransfection into Vero cells respectively. The transfection experiments were carried out by using established method in our laboratory [15,16,31]. Briefly, monolayers of Vero cells were transfected with indicated plasmid DNA mixed with Lipofectamine 2000 transfection reagent according to the manufacturer's instructions. At 18-20 h posttransfection (p.t.), cells were subjected to fluorescence microscopy observarion. Besides, for IF microscopy, transfected or infected cells were fixed for 20 min at room temperature (RT) with 4% paraformaldehyde in PBS, and then permeabilized with 0.2% TritonX-100 for 10 min. Permeabilized cells were blocked with PBS containing 3% bovine serum albumin (PBS-BSA) prior to incubation with primary VP6 specific antibody diluted in PBS-BSA. Hoechst staining was applied to detect the cell nucleus in this study. All the tested samples were observed using Olympus-IX51 inverted microscope equipped with phase and fluorescence optics. Collected images were processed with Pro-Eexpress 6.3 (Olympus) and Photoshop (Adobe Systems).

## Abbreviations

GCRV: Grass carp reovirus; Eagle’s MEM: Eagle’s minimum essential medium; DMEM: Dulbecco’s Modification of Eagle’s Medium; IF: Immunofluorescence; IB: immunoblotting; PVDF: polyvinylidene fluoride; NBT: nitrobluetetrazolium; BCIP: 5-bromo,4-chloro,3-indolylphosphate; AP: Alkaline phosphatase; RT: Room temperature.

## Competing interests

The authors declare that they have no competing interests.

## Authors’ contributions

QF designed the experiments. DW, LY carried out the experiments. LS, HG participated in partial work of recombinant plasmid constructions. QF, DW, LY and HG analyzed the data, QF, XL wrote the paper. All authors read and approved the final manuscript.
